# Investigating the mechanisms of drug resistance and prognosis in ovarian cancer using single-cell RNA sequencing and bulk RNA sequencing

**DOI:** 10.18632/aging.205628

**Published:** 2024-03-08

**Authors:** Pengfei Liu, Jinbao Liu, Jinxing Liu, Xiao Yu

**Affiliations:** 1 Shandong University of Traditional Chinese Medicine, Jinan, China; 2 Affiliated Hospital of Shandong University of Traditional Chinese Medicine, Jinan, China

**Keywords:** ovarian cancer, single-cell RNA sequencing, bulk RNA sequencing, resistance, prognosis

## Abstract

Ovarian cancer stands as a prevalent malignancy within the realm of gynecology, and the emergence of resistance to chemotherapeutic agents remains a pivotal impediment to both prognosis and treatment. Through a single-cell level investigation, we scrutinize the drug resistance and mitotic activity of the core tumor cells in ovarian cancer. Our study revisits the interrelationships and temporal trajectories of distinct epithelial cells (EPCs) subpopulations, while identifying genes associated with ovarian cancer prognosis. Notably, our findings establish a strong association between the drug resistance of EPCs and oxidative phosphorylation pathways. Subsequently, through subpopulation and temporal trajectory analysis, we confirm the intermediate position of EPCs subpopulation C0. Furthermore, we delve into the immunological functions and differentially expressed genes associated with the prognosis of C0, shedding light on the potential for constructing novel ovarian cancer prognosis models and identifying new therapeutic targets.

## INTRODUCTION

Ovarian cancer has exhibited a rising incidence globally in recent years, ranking as the most common and highly aggressive malignancy among gynecological tumors [1, 2]. Due to the lack of distinctive early symptoms, early detection through non-invasive means is challenging, resulting in a diagnosis at an advanced stage in up to 70% of ovarian cancer patients [3, 4]. According to global cancer statistics from 2020, there were 313,959 newly diagnosed cases of ovarian cancer worldwide, with 207,252 ovarian cancer-related deaths reported [5]. BRCA1 and BRCA2 stand out as widely recognized genetic susceptibility genes for ovarian cancer. Mutations in BRCA genes escalate the hereditary risk of ovarian cancer. Studies have indicated that females with mutations in the BRCA1 gene face a 3 to 4 times higher risk of developing ovarian cancer compared to those with mutations in the BRCA2 gene [6]. Like many cancer treatment modalities, surgery and chemotherapy are commonly employed in ovarian cancer management. However, the associated side effects and diminished quality of life often pose significant challenges for patients [7]. Furthermore, the high recurrence rates and development of drug resistance contribute to a 5-year survival rate of only approximately 47% for ovarian cancer patients [8]. Research has revealed that although most ovarian cancer patients initially respond to platinum-based chemotherapy, approximately 70-80% of patients eventually relapse and develop resistance, with the situation worsening over time [9–12]. In fact, the 5-year survival rate for advanced-stage high-grade serous ovarian cancer can be as low as 26% [13]. Presently, while neoadjuvant chemotherapy has emerged as a frontline treatment for certain patients, the impact of certain agents in neoadjuvant chemotherapy, such as bevacizumab, on the overall survival period of ovarian cancer, requires further investigation [14]. Additionally, there is a tendency for elevated drug resistance and recurrence rates with certain neoadjuvant chemotherapy approaches [15]. Targeted therapies for ovarian cancer have also shown limited success [16]. Immunotherapy primarily aims to disrupt immune suppression and tolerance states by enhancing the body’s immune recognition capabilities and immune-mediated tumor-killing abilities [17]. Some experts suggest that combining immunotherapy with PARP inhibitors and other treatment approaches can address the issue of tumor-infiltrating lymphocytes, but the effectiveness of such combinations still requires further research [18]. Due to the high heterogeneity of ovarian cancer, the development of immunotherapy has been relatively slow [19], and adverse treatment reactions have been reported intermittently [20]. In light of the diminished survival rates and heightened drug resistance observed in ovarian cancer recurrence, a thorough exploration of the mechanisms underpinning drug resistance in ovarian cancer becomes imperative. The search for novel therapeutic targets and immunotherapeutic strategies is crucial to enhance the efficacy of ovarian cancer treatment and to establish innovative prognostic models. In this study, we conducted a comprehensive investigation at the single-cell level to elucidate the mechanisms governing the extent of ovarian cancer’s response to drugs. Employing a subpopulation approach to epithelial cells (EPCs), we delineated the cellular trajectories and immune functionalities within relevant subgroups. Based on these research findings, we postulate a strong correlation between ovarian cancer drug resistance and the oxidative phosphorylation pathway, substantiated by our empirical evidence. These studies contribute to a more profound understanding of the distinctions between drug resistance and sensitivity at the cellular level in ovarian cancer, with the aim of informing the design of superior treatment targets and prognostic models for ovarian cancer.

## MATERIALS AND METHODS

### Data retrieval and processing

Single-cell samples from five cases of ovarian cancer were procured via the National Center for Biotechnology Information (NCBI) Gene Expression Omnibus (GEO) database (https://www.ncbi.nlm.nih.gov/geo/). The GSE number is GSE154600. These samples were identified as GSM4675273, GSM4675274, GSM4675275, GSM4675276, and GSM4675277. Gene expression quantification data derived from RNA-Seq (HTSeq TPM) and clinical information pertaining to ovarian cancer were sourced from The Cancer Genome Atlas (TCGA) platform (https://portal.gdc.cancer.gov). Subsequently, these datasets were subjected to rigorous standardization procedures using the R software environment (version 4.3.0).

### Data quality control

Utilizing the R package DoubletFinder, we meticulously filtered and excluded data from samples containing doublets and subpar cells. The filtration criteria encompassed: total gene transcription counts (nCount) per cell falling within the range of 500 to 80,000. Total number of genes detected per cell (nFeature) within the range of 200 to 6,000. Mitochondrial gene proportions lower than 25%. Red blood cell gene count proportions less than 5%.

### Dimension reduction, clustering, and annotation of data

Subsequent to filtering, we imported the single-cell data and performed normalization using the NormalizeData function from the Seurat package in the R environment [21, 22]. The FindVariableFeatures function was employed to compute the variance of each gene, enabling the selection of the top 2000 highly variable genes based on both their dispersion and mean expression [23, 24]. We centralized all genes using the ScaleData function, calculated cell cycle effects using the CellCycleFeatures function, and conducted dimensionality reduction through PCA on the top 2000 highly variable genes. Further, to mitigate batch effects originating from different samples, the RunHarmony function from the harmony package within R was deployed. Clustering was executed using the FindNeighbors and FindClusters functions from the Seurat package. Cell annotations were performed by cross-referencing available literature, the CellMarker database (http://bio-bigdata.hrbmu.edu.cn/CellMarker/), and SingleR, focusing on different cellular clusters. Differential gene expression between clusters was assessed via the FindAllMarkers function, with the top 5 marker genes being showcased.

### Drug resistance and staging analysis of entire cell population

Drug sensitivity among distinct cellular clusters in ovarian cancer was visually represented using UMAP visualization. Relationships between drug sensitivity and the relative proportions of various clusters were explored. Furthermore, employing the Ro/e algorithm, cell abundance under different drug sensitivity conditions was quantified. Similarly, using the aforementioned methodologies, cell staging in ovarian cancer clusters was calculated, including abundance and inter-cluster proportion relationships. To delve deeper into the relationship between cellular cluster staging and drug resistance, G2M and S phase scoring was computed for different ovarian cancer clusters, and their correlation with drug sensitivity was investigated.

### Enrichment analysis of entire cell population

Identification of differentially expressed genes (DEGs) between cellular clusters and other cell types was conducted. DEGs were considered significant if they were detected in at least 25% of cells with P < 0.01, a false discovery rate (FDR) < 0.05, and | logFCfilter | > 1. These DEGs were then subjected to Gene Ontology (GO) enrichment analysis. Furthermore, Gene Set Enrichment Analysis (GSEA) was performed, utilizing Kyoto Encyclopedia of Genes and Genomes (KEGG) databases (c2.cp.kegg.v7.5.1.symbols.gmt) to filter and analyze differentially expressed genes in Epithelial Cells (EPCs) compared to other clusters, with statistical significance defined as FDR < 0.05.

### Metabolism analysis of entire cell population

Employing the R package scMetabolism, we computed cellular metabolic pathways for different ovarian cancer clusters, stages, and drug resistance levels. The top 20 pathways were visually presented using a heatmap. We selected the top three pathways for in-depth exploration, investigating their expression patterns in UMAP representations and their associations with ovarian cancer clusters, drug sensitivity, and cell staging.

### EPCs subpopulation trajectory analysis

To investigate the transformation and evolution among subpopulations of EPCs, we conducted pseudo-time analysis. Using the R package Cytotrace, we predicted the relative differentiation status of each subpopulation of EPCs, with scores ranging from 0 to 1, where higher scores indicated a stronger cell stemness. Employing the R package monocle, we calculated the relative developmental time and sorted various subpopulations of EPCs accordingly. These cells were roughly categorized into three periods based on their sorting order. We explored the distribution of cell proportions among these three periods within each subpopulation of cells. Additionally, with respect to these three distinct states, we further investigated the temporal changes in cell cycle, subpopulation-specific genes, and marker genes. Genes were clustered into different groups based on their expression patterns over time, and we explored enriched pathways between these groups. Finally, we utilized slingshot to construct cell differentiation lineages and infer pseudo-time trajectories among different subpopulations of cells, incorporating both cell clustering and spatial dimension information.

### Gene and enrichment analysis of EPCs subpopulations

We explored genes related to subpopulations, especially those associated with cell stemness, and their expression changes over time. The expression distributions of four stemness-related genes significantly differing from other groups were visualized using UMAP. We employed the pyscenic algorithm to compute the top five transcription factors for both C0 and C2 subpopulations, investigating their overall distribution and regulatory regions within subpopulations. Furthermore, we calculated differential gene expression among subpopulations and performed GO and KEGG enrichment analyses. We also conducted GSEA enrichment analysis specifically for the C0 subpopulation.

### Clinical relevance and independent prognostic analysis of C0 subpopulation

Using R software, we identified intersection genes between C0 subpopulation marker genes and ovarian cancer tumor tissues and normal tissues. Incomplete clinical data for ovarian cancer samples were removed, and the intersection genes were merged with standardized clinical data for ovarian cancer. Cox proportional hazards regression analysis, including single-variable analysis, was performed using the coxph function from the survival package. Subsequently, we validated the results using least absolute shrinkage and selection operator (LASSO)-penalized Cox regression and obtained prognostic differential genes through multivariate Cox hazard regression analysis. Risk scores were calculated for each sample, where risk score = Xλ (relative expression levels of prognostic-related genes) and coefλ (coefficients) [25, 26]. Samples were categorized into high- and low-risk groups based on the median risk score, and Principal Component Analysis (PCA) was used to assess their distributions. Heatmaps were generated to visualize the expression patterns of prognostic genes in high- and low-risk groups. Additionally, we computed coxef values for prognostic genes, demonstrated the differential survival outcomes between high- and low-risk groups using Kaplan-Meier curves, and evaluated the prognostic specificity and sensitivity through time-dependent receiver operating characteristic (ROC) curves [27, 28]. Correlation analysis between genes and risk scores was also conducted. We incorporated patient age and ethnicity data to construct a nomogram using the rms package to predict the prognosis of ovarian cancer patients. The prognostic model was validated using overall survival (OS) and ROC curves.

### Immunological correlation analysis of C0 subpopulation

We calculated TIDE scores and major immune checkpoint scores for high- and low-risk groups. Additionally, we leveraged xCell and CIBERSORT deconvolution algorithms to assess the immune infiltration status of patients in the high- and low-risk groups. Correlations between immune cells, risk scores, and self-expression were investigated, and the relationships between M1 and M2 macrophages, T cells, and risk scores were explored based on the results.

### Enrichment analysis of prognostic genes

Using the R package limma, we calculated differential genes between high- and low-risk groups with filtering criteria of |log FC| > 1 and FDR (BH) corrected threshold P.adj < 0.05. We conducted KEGG enrichment analyses and Gene Ontology (GO) enrichment analyses using the “clusterProfiler” R package, which encompassed biological processes, cellular components, and molecular functions [29, 30]. GSEA enrichment analysis was also performed for differential genes between high- and low-risk groups [31, 32].

### Availability of data and material

The data for this study come from the Gene Expression Omnibus (GEO) (https://www.ncbi.nlm.nih.gov/geo/) database and The Cancer Genome Atlas database (https://portal.gdc.cancer.gov/). The GEO accession is GSE154600. All the data in this paper support the results of this study.

## RESULTS

### Ovarian cancer cell subpopulation classification

**Figure 1:**
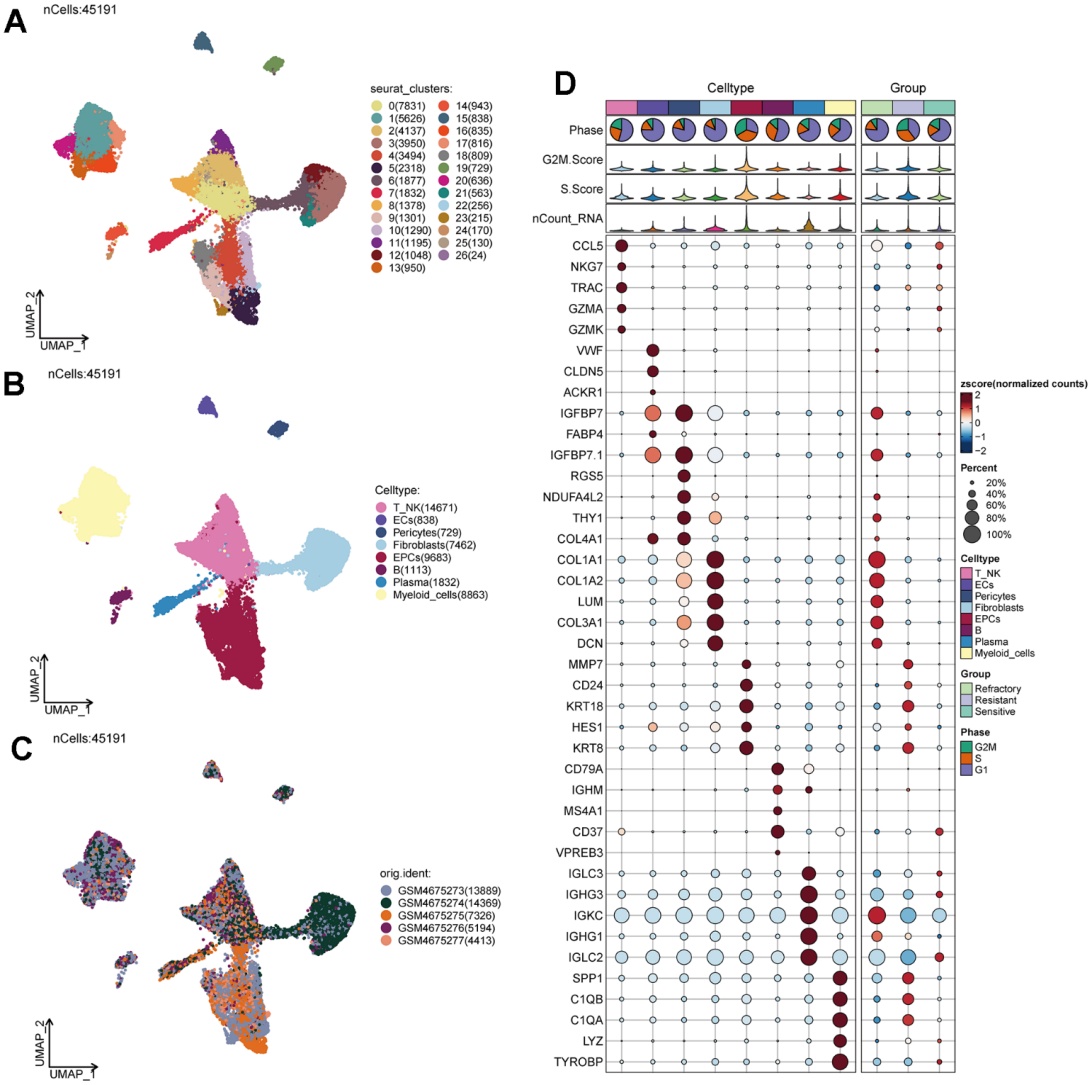
**Ovarian cancer cell cluster classification.** (**A**) A total of 28 clusters were identified after clustering 5 ovarian cancer patients. (**B**) Ovarian cancer cells were annotated into T_NK, ECs, Pericytes, Fibroblasts, EPCs, B Cells, Plasma, and Myeloid Cells based on distinct MARKER genes. (**C**) Distribution of cells from 5 different samples. (**D**) Expression patterns of the top 5 MARKER genes in each major cell cluster.

We obtained single-cell data from five ovarian cancer patients from the GEO database (GSM4675273-GSM4675277). We used the R package DoubletFinder to remove doublets from the samples (Supplementary Figure 1A–1E) and further filtered out low-quality single-cell data through R software, resulting in a clean dataset (Supplementary Figure 1F). After the removal of doublets, the number of cells was 46436. After the removal of low-quality cells, the number of cells was 45191. We observed that cells of different stages were relatively concentrated in the PCA plot (Supplementary Figure 1G), suggesting that cell staging had a minimal overall impact on our results. Based on gene expression and dispersion, we selected the top 2000 highly variable genes (Supplementary Figure 1H) and performed dimensionality reduction using RunPCA, choosing the top 30 dimensions for further analysis (Supplementary Figure 1I, 1J). We also explored the top ten highly variable genes for the first nine dimensions and presented them (Supplementary Figure 1K). Through clustering, we classified ovarian cancer cells into 27 clusters (Figure 1A) and further annotated them into eight major cell types using the R package singleR, Cellmarker database, and literature references (Figure 1B). These cell types included T_NK cells (14671), Endothelial Cells (ECs 838), Pericytes (729), Fibroblasts (7462), Epithelial Cells (EPCs 9683), B Cells (B 1113), Plasma Cells (1832), and Myeloid Cells (8863). Different samples showed variations in the distribution of cell types, possibly due to individual sample sources and the tissue of origin for the tumor cells (Figure 1C). We identified the top 5 MARKER genes for each of the eight major cell types and visualized them, along with G2M. Score, S.Score, and tumor treatment response (Figure 1D). We found that EPCs exhibited significantly elevated G2M.Score and S.Score, and their MARKER genes were highly correlated with drug resistance. Literature review revealed that EPCs are the primary tumor cells in ovarian cancer tissues, piquing our interest.

### Tumor treatment response and cell staging of EPCs

**Figure 2:**
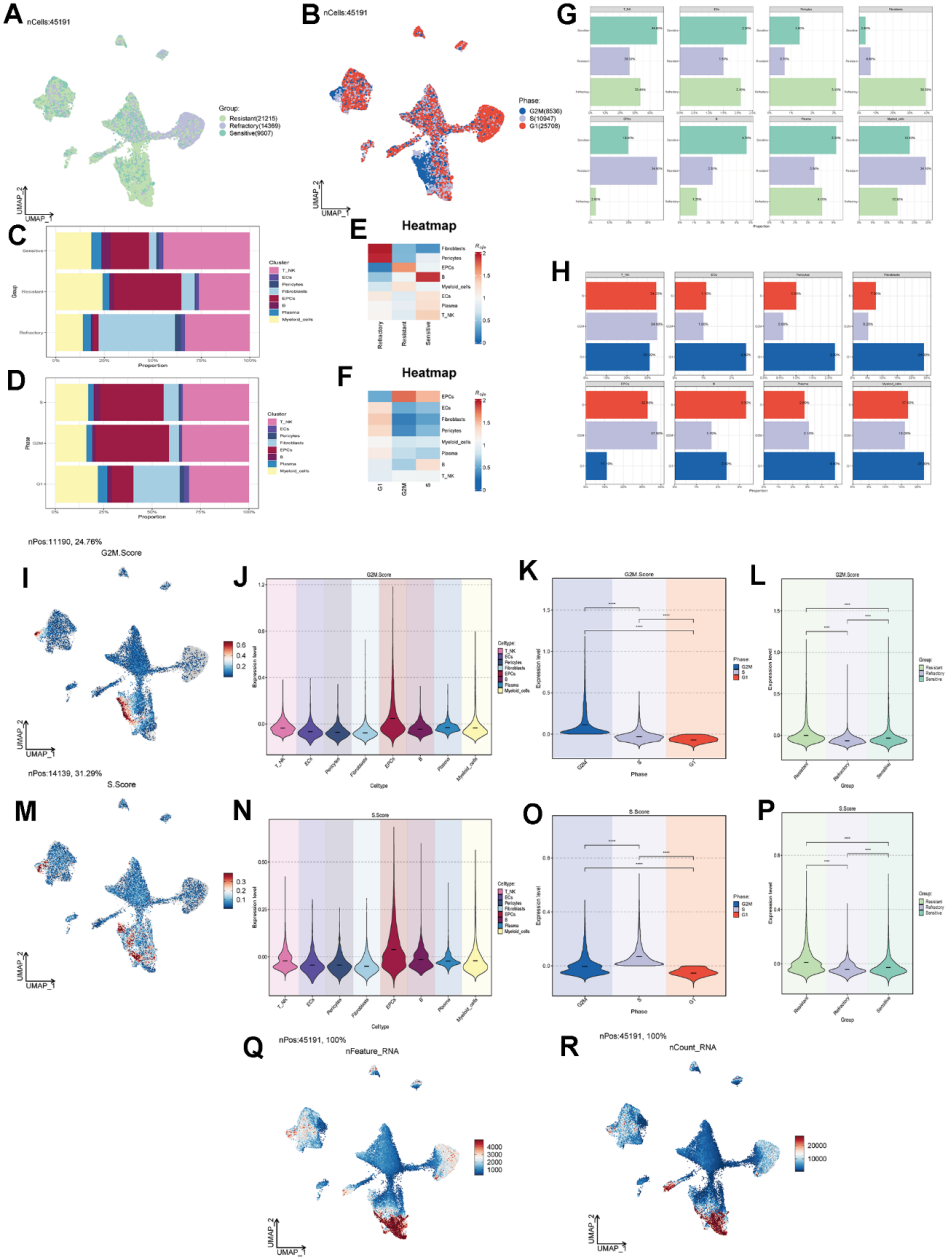
**Cell clustering by staging and drug treatment response level.** (**A**) Distribution of UNMP drug treatment response levels. (**B**) Distribution of UNMP cell staging within major cell clusters. (**C**) Proportion of major cell clusters within the Resistant, Refractory, and Sensitive response groups. (**D**) Proportion of major cell clusters within the G2M, S, and G1 cell staging phases. (**E**, **F**) Abundance of major cell clusters concerning drug response groups and cell staging. (**G**) Proportion of major cell clusters within the Resistant, Refractory, and Sensitive response groups and their facets were shown. (**H**) Proportion of major cell clusters within the G2M, S, and G1 cell staging phases and their facets were shown. (**I**) Distribution of UNMP G2M.Score. (**J**–**L**) Violin plots for G2M.Score in major ovarian cancer cell clusters, cell staging, and drug response. (**M**) Distribution of UNMP S.Score. (**N**–**P**) Violin plots for S.Score in major ovarian cancer cell clusters, cell staging, and drug response. (**Q**, **R**) Distribution of UNMP nFeature and nCount.

Tumor treatment response can be categorized into three groups: Resistant, Refractory, and Sensitive. We studied the treatment response of various cell types in ovarian cancer tissues (Figure 2A) and found that the Resistant group was predominantly located within the EPCs cluster. Similarly, we observed that the G1 phase is a period after cell division and before DNA replication, the S phase is dedicated to DNA replication, and the G2M phase is the cell division preparation and mitotic phase. Abnormalities in these stages can lead to tumorigenesis. Combining cell staging with our findings, we noted that the G2M and S stages were significantly concentrated within the EPCs cluster (Figure 2B). To gain a more intuitive understanding of the distribution of treatment responses, we examined the proportions of different cell types in ovarian cancer (Figure 2C, 2G) and found that the Resistant group had the highest proportion of EPCs. Regarding tumor staging (Figure 2D, 2H), the proportion of EPCs in the G2M and S stages was significantly higher than in other cell types, while the G1 stage proportion was relatively lower. We also investigated the cell abundance of different cell groups in ovarian cancer, based on treatment response and cell cycle (Figure 2E, 2F). We observed that the Resistant group had the highest cell purity of EPCs. Similarly, in the G2M and S phases, the purity of EPCs was much higher than that of other types of ovarian cancer cells. This suggests that EPCs are highly resistant to treatment and exhibit more vigorous cellular activity, and there may be some underlying connections between these characteristics. Furthermore, we validated our findings using G2M.Score and S.Score, which showed that EPCs had significantly higher scores compared to other cell types, with the Resistant group exhibiting the highest scores among all groups (Figure 2I–2P). We also examined the number and types of DNA replications among different cell groups (Figure 2Q, 2R) and found that EPCs exhibited significantly higher scores. This might be related to the robust proliferative capacity of tumor cells.

### Enrichment functional analysis of ovarian cancer cell subpopulations

**Figure 3:**
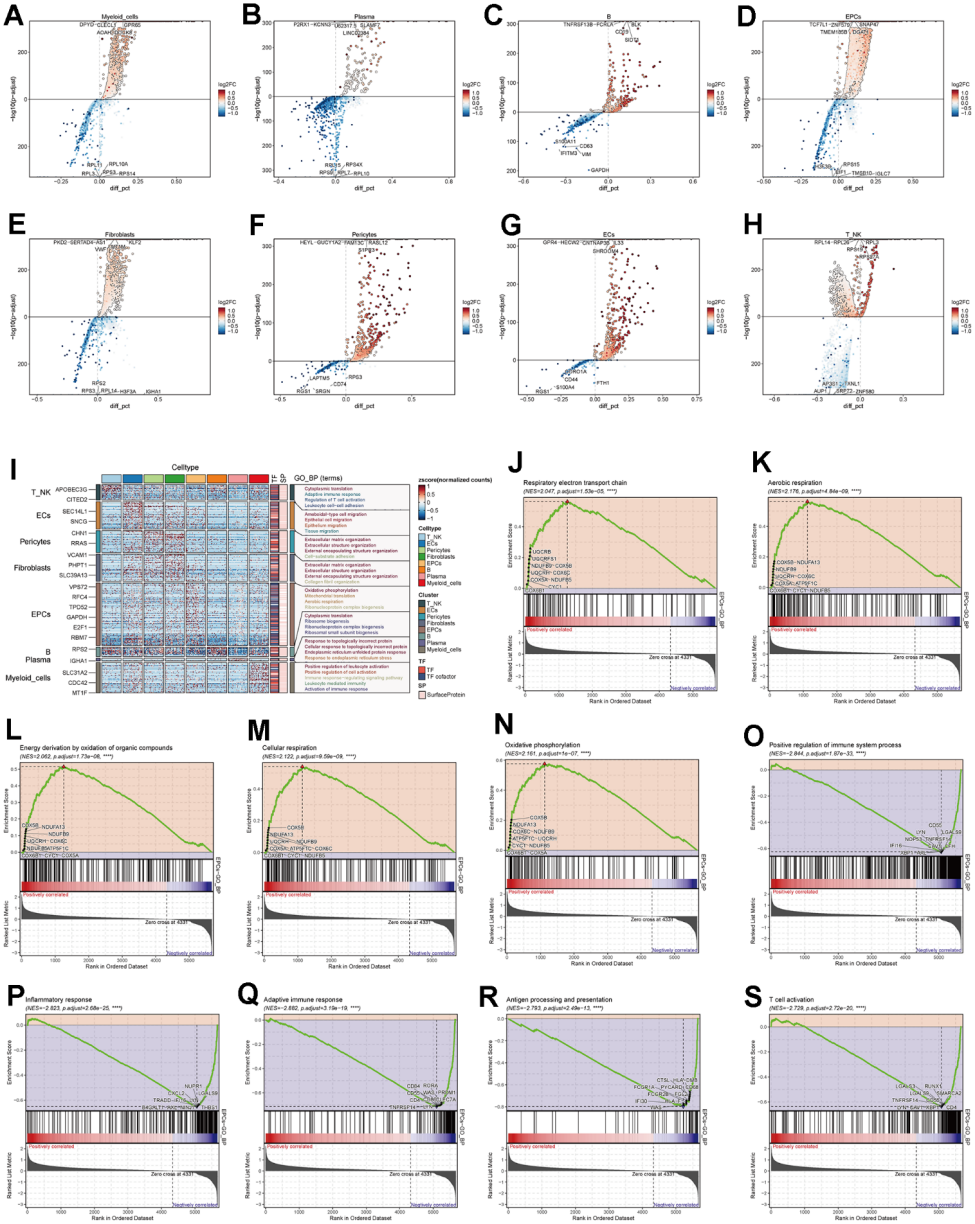
**Enrichment analysis of major cell clusters.** (**A**–**H**) Differential gene distribution in major ovarian cancer cell clusters compared to other cell types, displaying the top 5 upregulated and downregulated genes for each. (**I**) GO enrichment analysis for major cell clusters. (**J**–**S**) GSEA enrichment analysis for EPCs, displaying 5 pathways selected from both low and high expression groups.

We conducted differential gene expression analysis among various cell types in ovarian cancer tissues and presented the top 5 most significant genes (Figure 3A–3H). Using these differential genes, we performed GO enrichment functional analysis (Figure 3I) and found significant enrichment of pathways such as “Oxidative phosphorylation,” “Mitochondrial translation,” and “Aerobic respiration” in EPCs. Through GSEA enrichment functional analysis (Figure 3J–3S), we observed significant enrichment of pathways such as “Oxidative phosphorylation,” “Energy derivation by oxidation of organic compounds,” “Respiratory electron transport chain,” “Aerobic respiration,” and “Cellular respiration” in the high-expression group. In contrast, pathways including “T cell activation,” “Adaptive immune response,” “Inflammatory response,” “Positive regulation of the immune system process,” and “Antigen processing and presentation” were significantly enriched in the low-expression group. Oxygen-related pathways were prominently enriched in both GO and GSEA pathways. To further explore the underlying mechanisms, we conducted cellular metabolism analysis.

### Metabolism-related analysis of ovarian cancer cell subpopulations

**Figure 4:**
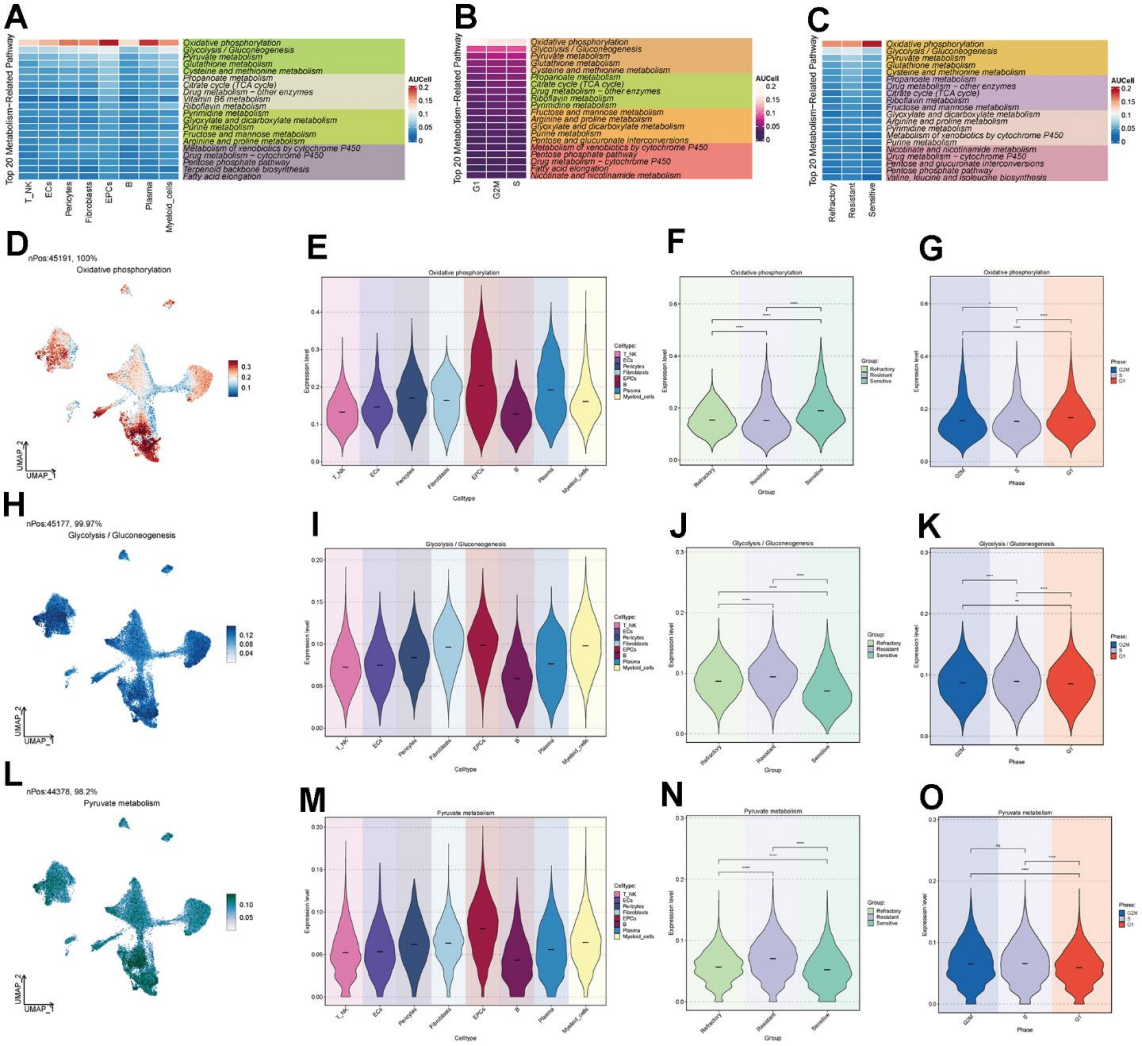
**Metabolic pathways of major cell clusters.** (**A**) Top 20 metabolic pathways in major ovarian cancer cell clusters. (**B**) Top 20 metabolic pathways in G2M, S, and G1 cell staging. (**C**) Top 20 metabolic pathways in the Resistant, Refractory, and Sensitive response groups. (**D**–**O**) UNMP distribution and violin plots for the Oxidative Phosphorylation, Glycolysis / Gluconeogenesis, and Pyruvate Metabolism pathways in major ovarian cancer cell clusters, cell staging, and drug response.

Through cellular metabolism analysis (Figure 4A–4C), we found that Oxidative Phosphorylation, Glycolysis/Gluconeogenesis, and Pyruvate Metabolism scored significantly in various ovarian cancer cell subpopulations, treatment response levels, and cell staging. Among them, Oxidative Phosphorylation had a higher score in EPCs (Figure 4D–4G), suggesting that this metabolic pathway might be a key pathway in EPCs’ metabolism. The other two metabolic pathways, Glycolysis/Gluconeogenesis and Pyruvate Metabolism (Figure 4H–4O), scored higher not only in the EPCs group but also in the Resistant group and in cells in the G2M and S phases. We speculate that these two pathways may play a positive role in tumor progression.

### Subpopulations of EPCs

**Figure 5:**
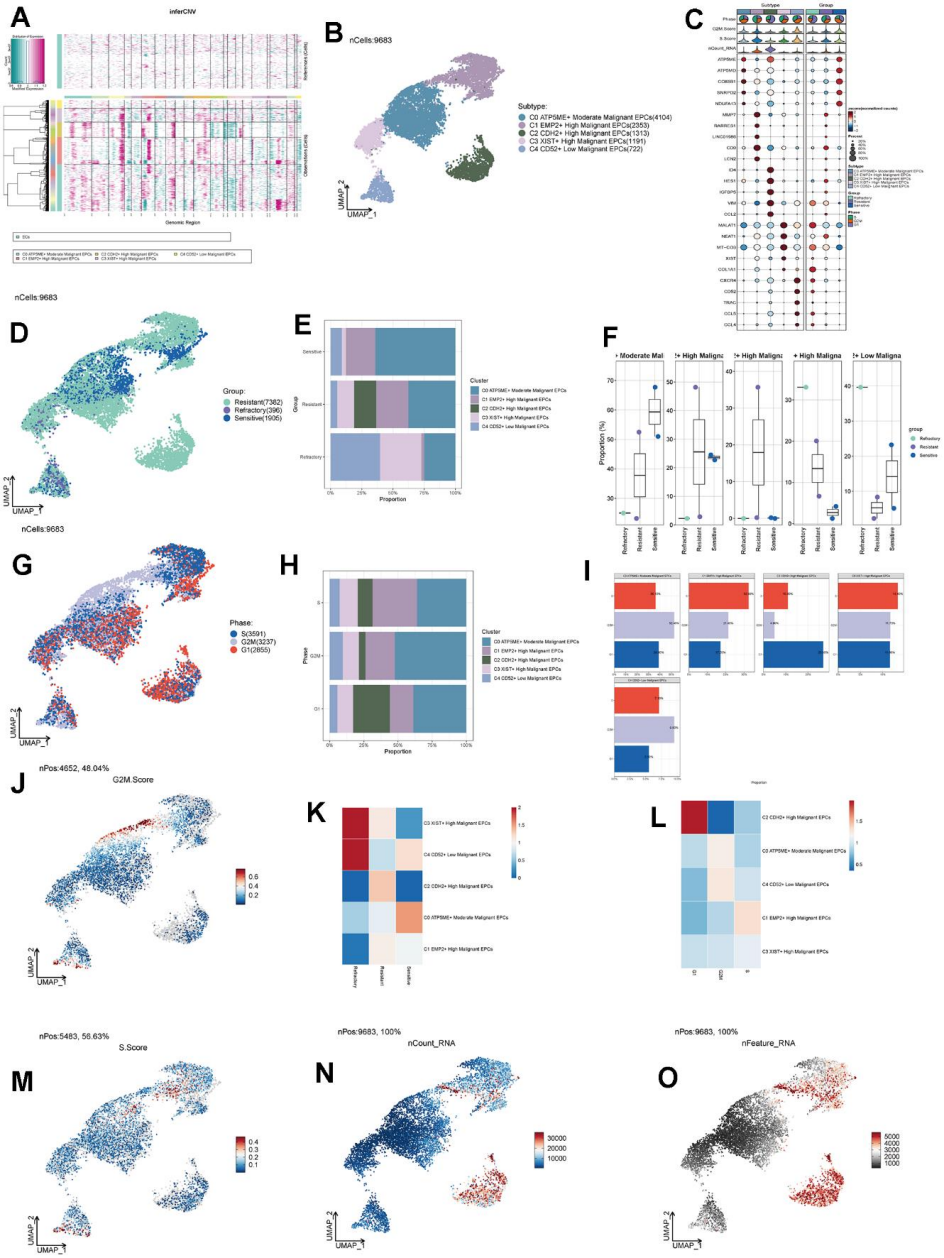
**Subgroup analysis of EPCs and related data.** (**A**) Heatmap showing CNV status of subgroup cells. (**B**) UNMP distribution of subgroup cells. (**C**) Expression patterns of the top 5 signature genes in each subgroup. (**D**) UNMP distribution of drug responses in subgroup cells. (**E**) Proportion of subgroup cells within the Resistant, Refractory, and Sensitive response groups. (**F**) Proportion of subgroup cells within the Resistant, Refractory, and Sensitive response groups and their facets were shown. (**G**) UNMP distribution of cell staging in subgroup cells. (**H**) Proportion of subgroup cells within the G2M, S, and G1 cell staging phases. (**I**) Proportion of subgroup cells within the G2M, S, and G1 cell staging phases and their facets were shown. (**J**) UNMP distribution of G2M.Score. (**K**, **L**) Abundance of subgroup cells concerning drug response groups and cell staging. (**M**–**O**) UNMP distribution of S.Score, nFeature, and nCount.

To further study the composition and functions of EPCs, we re-clustered EPCs into five subpopulations (C0-C4). Based on the calculation of each subpopulation’s CNV (Figure 5A), we labeled the C4 group with the lowest CNV as Low Malignant EPCs, and the C1, C2, and C3 groups with the highest CNV as High Malignant EPCs. The intermediate group, C0, was labeled as Moderate Malignant EPCs. We selected a signature gene for each subpopulation based on the expression level and percentage of MARKER genes (Figure 5B). We also investigated the top 5 MARKER genes for each subpopulation, as well as their relationship with cell staging and treatment response levels (Figure 5C). We found that in EPCs, the expression of C0 subpopulation’s MARKER genes highly overlapped with the Sensitive group and was strongly correlated with G2M.Score and S.Score. This seemed contrary to the characteristics of the overall EPCs population being Resistant, which piqued our interest. We further explored the drug treatment response of each subpopulation (Figure 5D) and found that the C0 group exhibited higher drug sensitivity, while the C2 group showed higher drug resistance. Moreover, we observed the proportions of each subpopulation (Figure 5E, 5F), with C0 having the highest proportion of Sensitive cells, while C2 had the highest proportion of Resistant cells, and the C1 group showed a distribution between the two. Cell abundance calculations were consistent with these findings, with C0 having the highest purity in the Sensitive group, and C2 having the highest purity in the Resistant group (Figure 5K). We also calculated cell staging and proportions for each subpopulation (Figure 5G–5I) and found that in the cell cycle distribution, the G1 phase was most prevalent in C2, while the G2M and S phases were more common in C0 and C1 subpopulations. Cell abundance calculations corresponded to these results (Figure 5L). We further calculated G2M.Score, S.Score, nFeature, and nCount values for each subpopulation, finding that C0 and C1 subpopulations had higher proliferative activities (Figure 5J, 5M), while C2 had relatively higher metabolic activities (Figure 5N, 5O). We believe that C0 may represent a subpopulation of drug-sensitive cells within the overall Resistant EPCs population. In-depth exploration of the causes and mechanisms of drug sensitivity is crucial for ovarian cancer treatment.

### Pseudotime analysis of subpopulation cells

**Figure 6:**
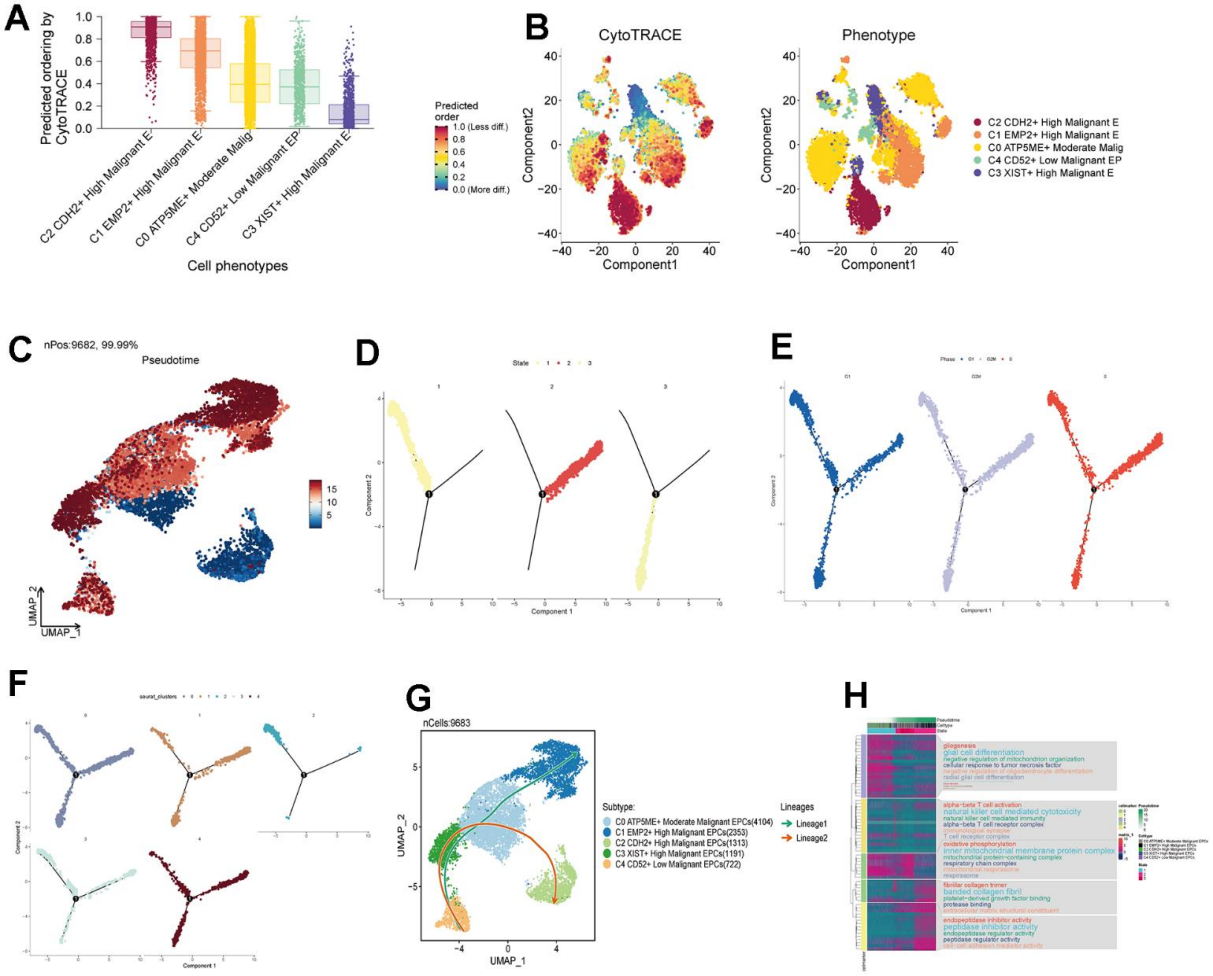
**Pseudotemporal analysis of subgroup cells.** (**A**, **B**) Assessment of Cytotrace scores in subgroup cells. (**C**) Pseudotemporal chart of subgroup cell UNMP based on Monocle analysis. (**D**) Temporal distribution of 3 states, with time progressing from State 1 to State 3. (**E**) Temporal distribution of subgroup cell staging. (**F**) Pseudotemporal distribution chart of subgroup cells. (**G**) Time trajectory of subgroup cells based on Slingshot analysis. (**H**) Enrichment analysis pathways based on the order of gene expression.

We predicted the differentiation status of each subpopulation using Cytotrace and found that the C2 group had the lowest differentiation, followed by C1 and C0 (Figure 6A, 6B). To understand the temporal relationships between subpopulation cells, we calculated the relative temporal order of each subpopulation using monocle (Figure 6C) and divided all subpopulations into three time stages: State1 as the initial stage, State2 as the intermediate stage, and State3 as the terminal stage (Figure 6D). We found that G1, G2M, and S stages were present in all three states (Figure 6E), but C2 was almost exclusively in State1, while C0 was more prevalent in State2 (Figure 6F). We speculate that the C2 group is at the starting point of the trajectory, while C0 is at the center of the trajectory and progresses toward the surrounding subpopulations. We further validated the cell trajectory using slingshot and confirmed that C0 indeed occupies the intersection of the cellular trajectory (Figure 6G). This aligns with our previous research findings. Finally, we performed GO enrichment analysis based on the different gene expression order within each subpopulation and found that oxidative phosphorylation pathways were significantly enriched in the initial and intermediate stages, suggesting that this pathway plays a crucial role in the regulation of drug response, leading to the development of drug resistance and sensitivity (Figure 6H).

### Functional analysis of subpopulation cells

**Figure 7:**
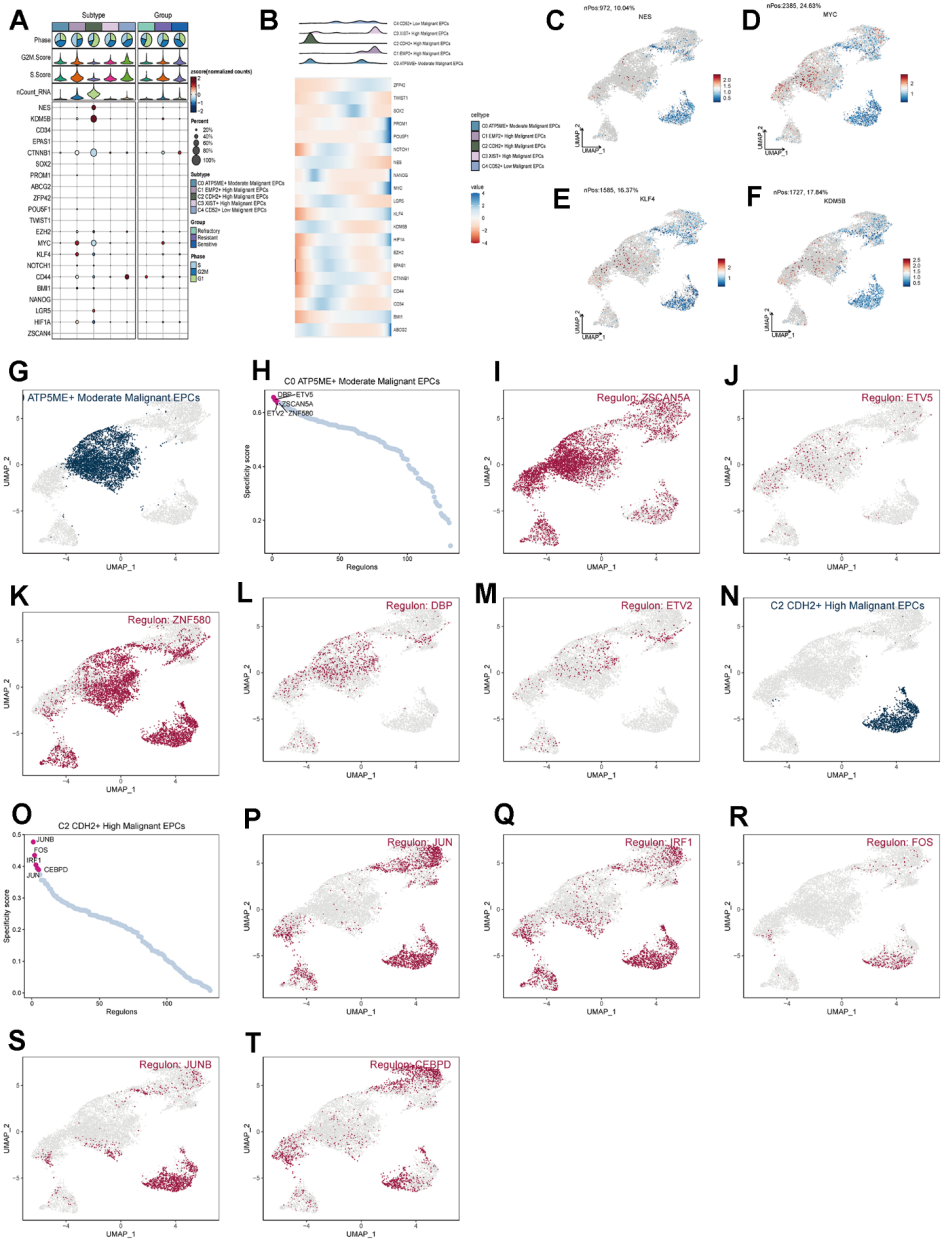
**Analysis of subgroup cell stemness genes and transcription factors.** (**A**) Expression patterns of stemness genes in various subgroups. (**B**) Temporal order of stemness gene expression in subgroup cells. (**C**–**F**) Distribution of NES, KDM5B, KLF4, and MYC gene UNMP expression. (**D**–**T**) Distribution of UNMP for the top five transcription factors in both C0 and C2 and their regulatory regions.

**Figure 8:**
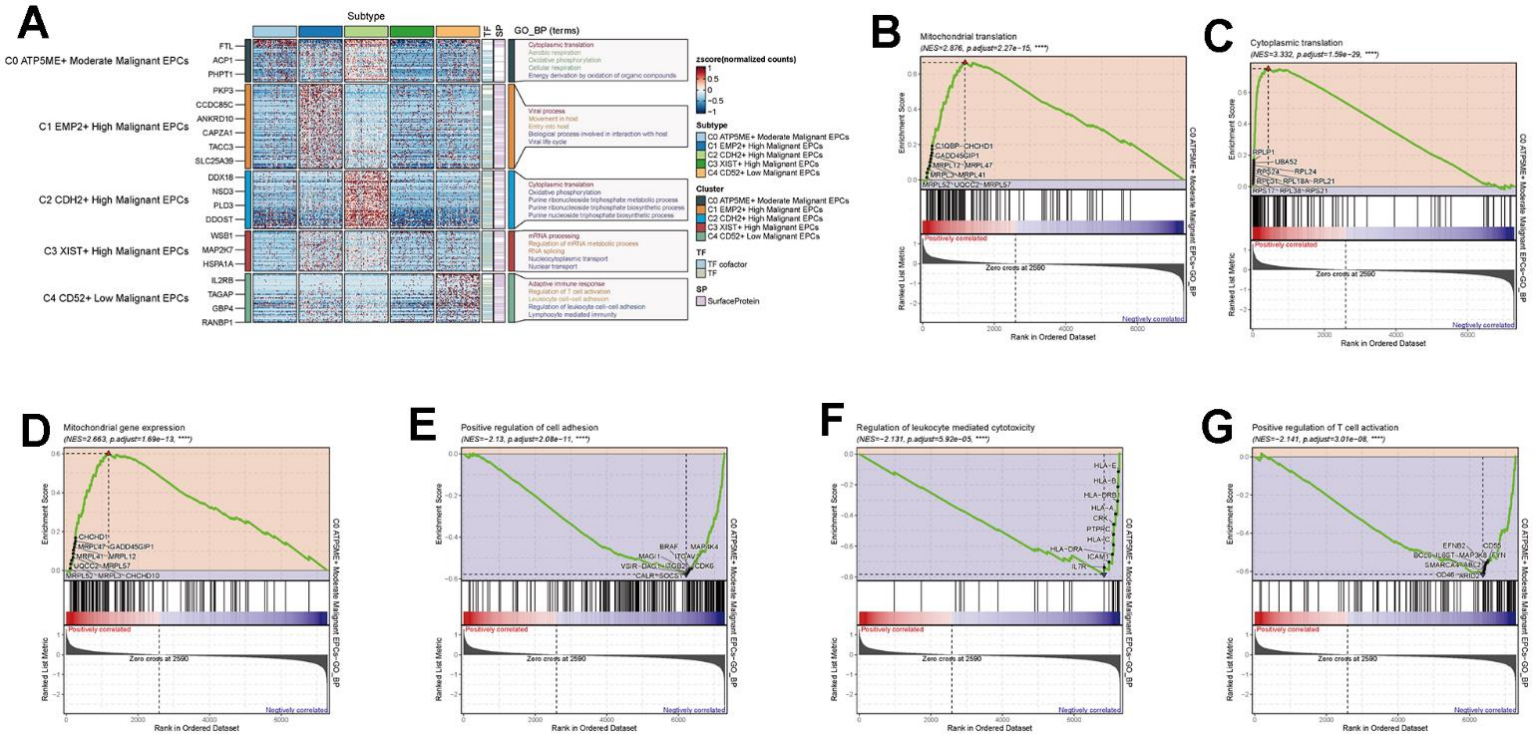
**Enrichment analysis of subgroup cells.** (**A**) GO enrichment analysis of subgroup cells. (**B**–**G**) GSEA enrichment analysis of C0 subgroup cells.

We calculated the core genes and their expression changes over time in each subpopulation (Figure 7A, 7B). We observed that NES and KDM5B were significantly expressed in the C2 group and decreased with time, while the genes KLF4 and MYC increased with time (Figure 7C–7F). In addition, to study the relationship between the top five transcription factors in C0 and C2 and their regulatory regions, we analyzed them (Figure 7G–7T) and found that the transcription factor ZNF580 played a significant regulatory role not only in the C0 subpopulation but also in the C2 subpopulation. By conducting GO enrichment analysis for subpopulation cells (Figure 8A), we found that oxidative phosphorylation pathways were significantly enriched in both C0 and C2 subpopulations, with a more concentrated presence of oxygen-related and cellular respiration pathways in C0. Based on this, we conducted GSEA enrichment analysis for the C0 group and found significant enrichment of pathways such as “Cytoplasmic Translation,” “Mitochondrial Gene Expression,” and “Mitochondrial Translation” in the high-expression group (Figure 8B–8D). Conversely, pathways like “Positive Regulation of T Cell Activation,” “Regulation of Leukocyte-Mediated Cytotoxicity,” and “Positive Regulation of Cell Adhesion” were significantly enriched in the low-expression group (Figure 8E–8G). This suggests that the C0 group is indeed associated with certain oxidative and respiratory functions.

### Prognostic analysis

**Figure 9:**
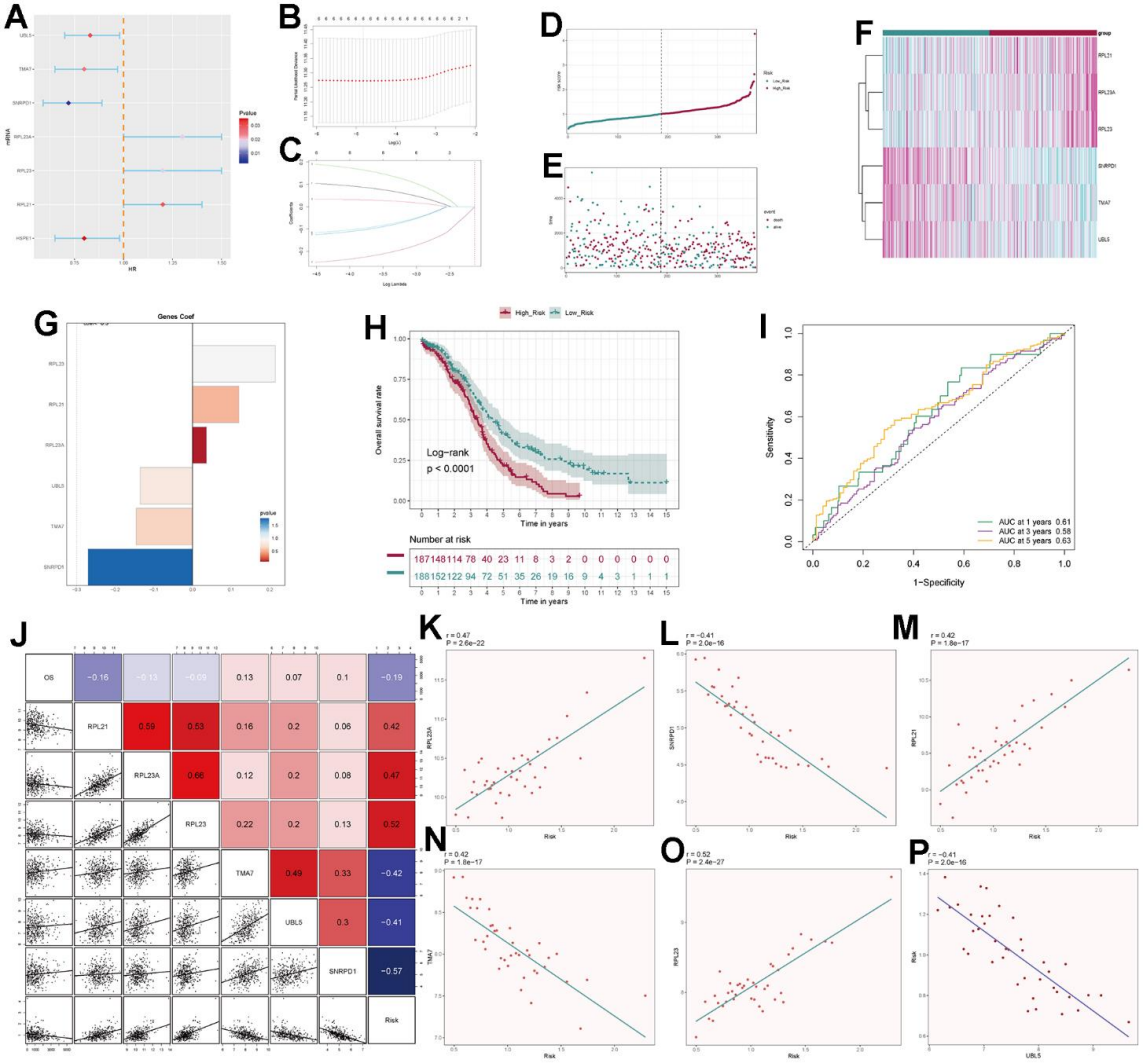
**Independent prognostic analysis.** (**A**) Seven differentially expressed genes associated with prognosis. (**B**) Distribution of LASSO analysis coefficient spectrum for six prognostic genes. (**C**) Parameter selection in the optimal cross-validation LASSO regression. (**D**) Patients categorized into high- and low-risk groups based on their risk scores. (**E**) Distribution of patients in the high- and low-risk groups. (**F**) Heatmap showing the distribution of prognosis-related genes. (**G**) Cofe values of prognosis-related genes. (**H**) Kaplan-Meier survival analysis curves for high- and low-risk groups. (**I**) Time-dependent ROC curves with area under the curve (AUC) values of 0.61, 0.58, and 0.63 for 1-year, 3-year, and 5-year intervals. (**J**–**P**) Correlation analysis of genes with risk scores and overall survival (OS).

**Figure 10:**
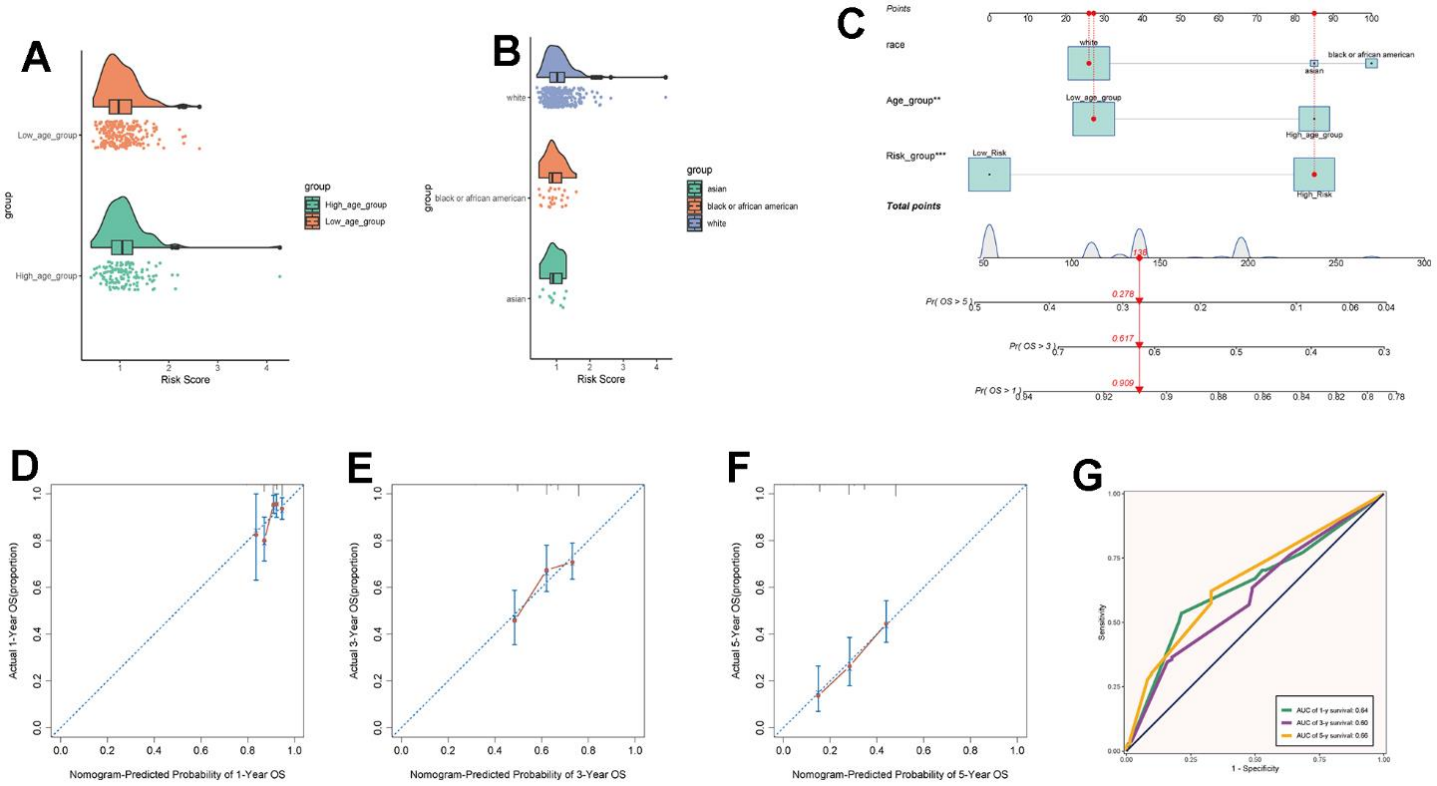
**Clinical relevance analysis.** (**A**, **B**) Analysis of the correlation between risk scores and age as well as ethnicity factors. (**C**) Survival curves for ovarian cancer patients at 1-year, 3-year, and 5-year intervals. (**D**–**F**) Nomo model charts for 1-year, 3-year, and 5-year OS tests. (**G**) Time-dependent ROC curve graphs with AUC values of 0.64, 0.60, and 0.66 for 1-year, 3-year, and 5-year intervals, respectively.

By intersecting the differential genes between ovarian cancer and C0 subpopulation signature genes in the TCGA database and using univariate Cox regression analysis, we identified 7 genes related to prognosis (Figure 9A). We verified these genes using LASSO Cox regression analysis, which showed stable and good performance (Figure 9B, 9C). Finally, through multivariate Cox regression analysis, we determined 6 prognostic genes, where RPL21, RPL23A, and RPL23 were high-risk genes, while SNRPD1, TMA7, and UBL5 were low-risk genes. We calculated the risk scores for each sample and divided them into high and low-risk groups based on the median score (Figure 9D). We assessed the relationship between the high and low-risk groups in terms of survival status and survival time (Figure 9E) and found that as the risk score increased, the number of deceased patients became more concentrated. We further studied the expression of prognostic genes in the high and low-risk groups (Figure 9F) and examined the coefficient values of prognostic genes (Figure 9G). Among them, SNRPD1 had the lowest coefficient, while RPL23 had the highest. Kaplan-Meier curves (Figure 9H) showed that the high-risk group had lower survival rates at different time points, with p < 0.001, indicating meaningful results. The ROC curve results for 1-year, 3-year, and 5-year survival were 0.61, 0.58, and 0.63, respectively (Figure 9I), indicating stable and excellent predictive performance. We further explored the correlation between prognostic genes, risk scores, and OS (Figure 9J–9P). The results were consistent with our previous research. We also investigated the correlation between different clinical factors and risk scores (Figure 10A, 10B) and constructed nomogram plots based on risk scores and clinical factors to predict the 1-year, 3-year, and 5-year survival rates of different patients (Figure 10C). We validated the accuracy of the 1-year, 3-year, and 5-year prognostic models (Figure 10D–10F). We also plotted ROC curves for each year’s survival (Figure 10G), with AUC values of 0.64, 0.60, and 0.66 for 1-year, 3-year, and 5-year survival, respectively, indicating good sensitivity and specificity and excellent predictive value.

### Immune-related analysis and enrichment analysis

**Figure 11:**
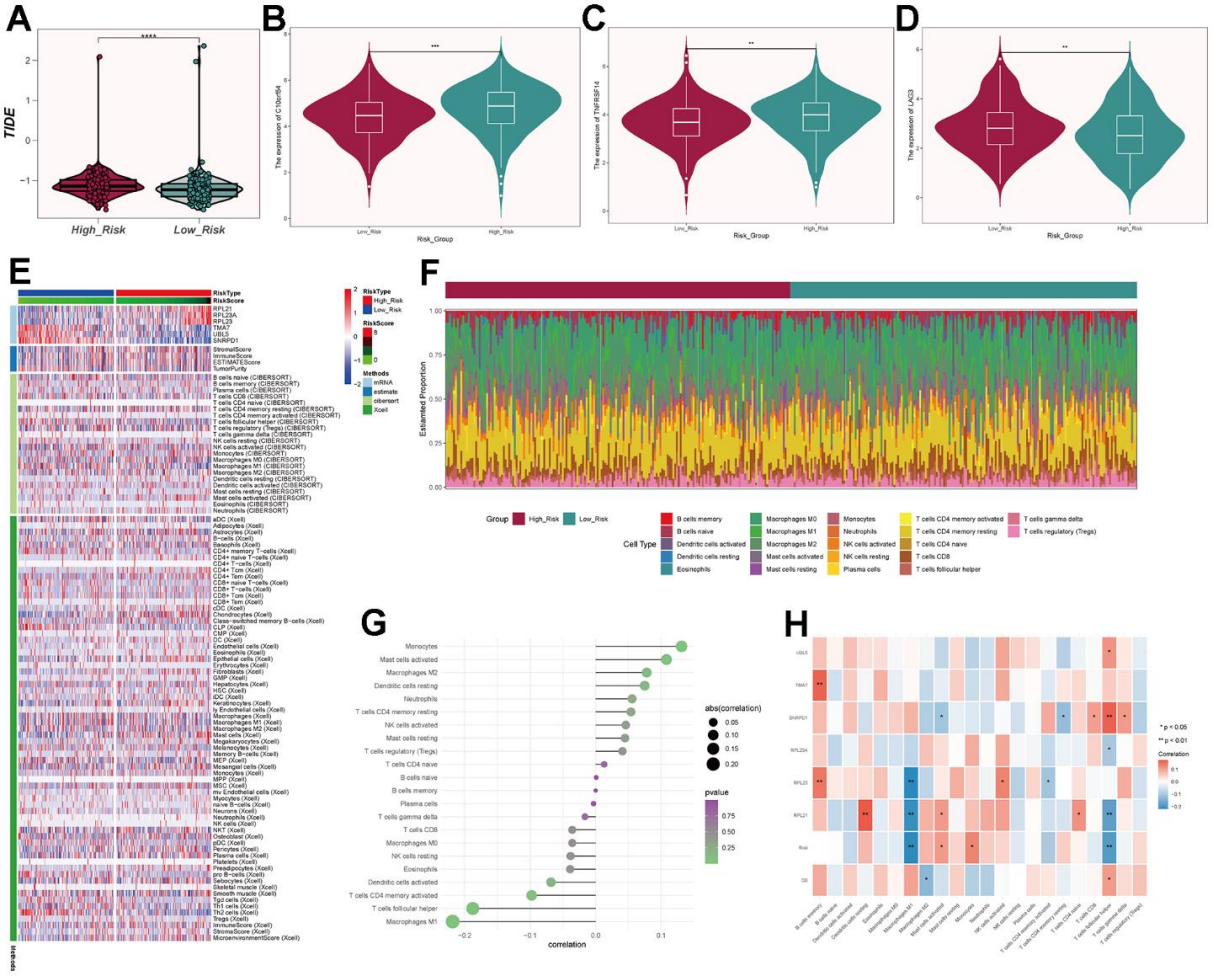
**Immune-related analysis of C0 subgroup.** (**A**) Violin plot of TIDE scores for high- and low-risk groups. (**B**–**D**) Immune checkpoint scores for high- and low-risk groups. (**E**) Heatmap showing differences in predictive genes, tumor microenvironment, and immune cells between high- and low-risk groups. (**F**) Proportion of immune cells in high- and low-risk groups. (**G**) Correlation between immune cells and risk scores. (**H**) Correlation between immune cells, prognosis-related genes, risk scores, and overall survival (OS).

**Figure 12:**
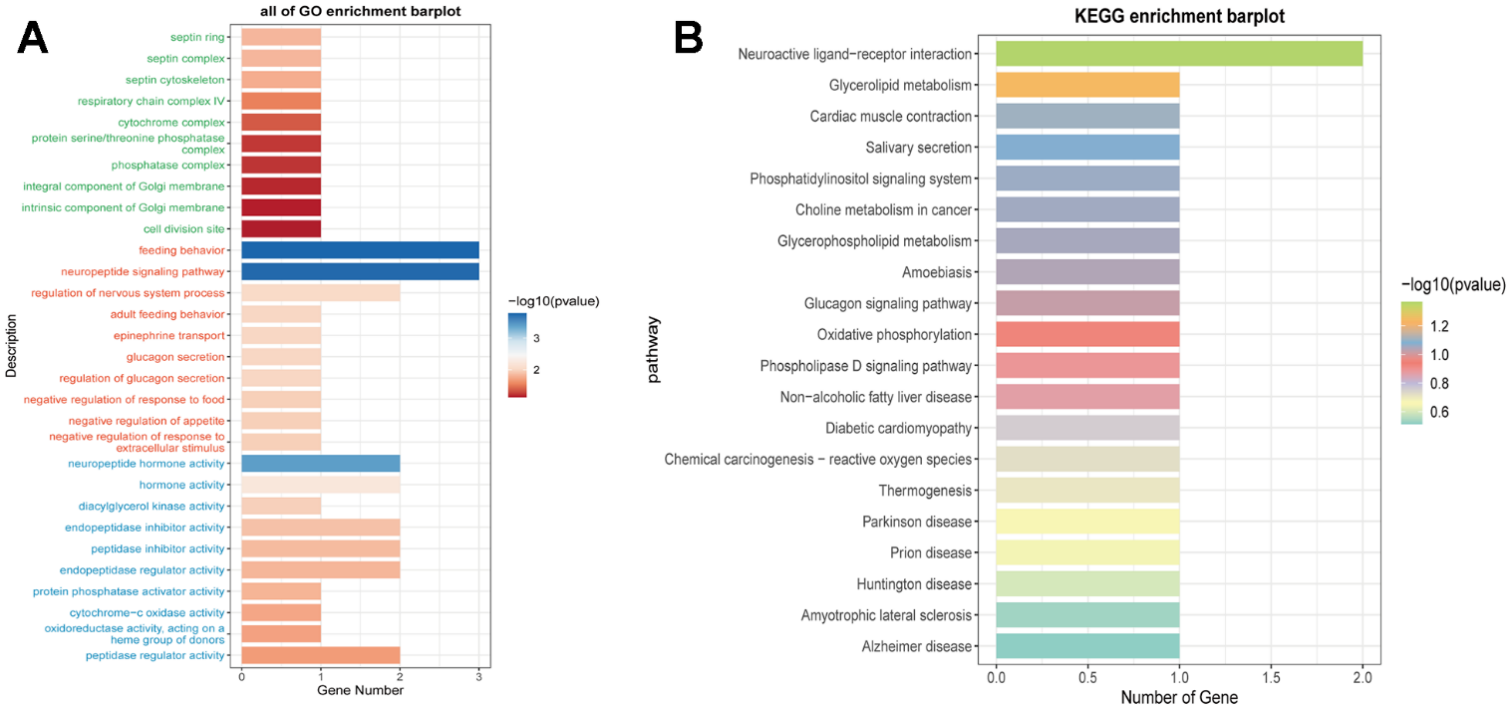
**Enrichment analysis of prognostic genes.** (**A**) Functional enrichment analysis of prognostic genes using GO terms. (**B**) Functional enrichment analysis of prognostic genes using KEGG pathways.

We calculated the TIDE scores and found that the high-risk group had higher scores (Figure 11A), indicating a higher likelihood of immune escape in high-risk group tumors. We screened immune checkpoint genes in the high and low-risk groups and found that TNFRSF14 and C10orf54 were significantly expressed in the low-risk group, while LAG3 was expressed at higher levels in the high-risk group (Figure 11B–11D). To further study the immune relevance of high and low-risk groups, we calculated immune cell infiltration (Figure 11E, 11F) and explored the relationships between risk scores, immune cells, and prognostic genes (Figure 11G, 11H). We found that M1 macrophages were negatively correlated with risk scores and RPL23, RPL21, while M2 macrophages were positively correlated with risk scores. Furthermore, T cells follicular helper exhibited strong correlations with SNRPD1 and risk scores. We hypothesized that M1 macrophages and T cells follicular helper were crucial for prognosis and immunotherapy, while M2 macrophages played an opposing role in this context. We also performed GO and KEGG enrichment analysis based on the differential genes between high and low-risk groups (Figure 12A, 12B) and found significant enrichment of pathways such as “Feeding Behavior” and “Neuropeptide Signaling Pathway” in GO, and “Neuroactive Ligand−Receptor Interaction" and “Glycerolipid Metabolism” in KEGG.

## DISCUSSION

Ovarian cancer stands as one of the three most prevalent malignancies within the female reproductive system. According to data from the United States in 2020, newly diagnosed cases of ovarian cancer accounted for 2.4% of all new female cancer cases and contributed to 5% of female cancer-related mortalities [5]. The persistent challenge of platinum resistance and the consequent recurrence have consistently led to a low 5-year survival rate among ovarian cancer patients [33]. Presently, our understanding of the mechanisms governing tumor resistance and sensitivity remains relatively limited [34]. Hence, the pursuit of the underlying mechanisms that contribute to tumor responsiveness and resistance assumes paramount importance. In our study, we observed that the Oxidative Phosphorylation pathway scored notably high in both enrichment and metabolic analyses within the tumor core cells, specifically EPCs. This high score was also evident in the crucial subpopulation C0. We postulate that this pathway might be involved in the mechanisms of tumor drug resistance and certain other aspects of progression. Notably, this pathway exhibits a close association with EPC subtypes, particularly C0 and C2. Prior studies have underscored the pivotal role of oxidative phosphorylation in sustaining the vital processes of certain cancers [35–37]. It fulfills the tumor’s energy and metabolite requirements, thereby fostering its growth [38]. Remarkably in ovarian cancer, research has also identified the upregulation of gene activity in the oxidative phosphorylation pathway within ovarian cancer tumor cells that are involved in chemotherapy resistance [39]. Moreover, the application of oxidative phosphorylation inhibitors has been found to effectively inhibit the progression of ovarian cancer [40]. Tang et al. observed that inhibiting the oxidative phosphorylation pathway with metformin resulted in a reduction in tumor growth and migration, accompanied by an increase in apoptosis [41]. Our research corroborates these findings by revealing a high correlation between the oxidative phosphorylation pathway and EPCs, a subset exhibiting the highest degree of resistance within the larger cell population.

Matassa and colleagues have similarly suggested the involvement of the oxidative phosphorylation pathway in ovarian cancer cell resistance, possibly mediated through TRAP1 [42]. Our investigations have additionally exposed disparities between subtypes C0 and C2, hinting that targeted intervention within the oxidative phosphorylation pathway may induce alterations in the proportions of EPC subtypes. This intervention could potentially prompt a transition from C2 to C0 and other subtypes, thereby ameliorating drug resistance in ovarian cancer tumor cells.

We have further discerned that the C0 EPC subtype exhibits a suppression of both immune infiltration and immune regulatory functions. Intriguingly, the correlation between macrophages M1 and M2 and risk scores runs contrary to expectations. Tumor-associated macrophages (TAMs) constitute a crucial component of the tumor microenvironment, infiltrating to levels exceeding 50%. They play a significant regulatory role in angiogenesis, tumor growth, and metastasis [43, 44]. TAMs can be specifically categorized into M1 and M2 macrophages. In the early stages of tumor development, TAMs exhibit a pro-inflammatory phenotype similar to M1, suppressing the immune response against tumor growth. As the tumor progresses, the hypoxic conditions within the tumor microenvironment gradually induce the transformation of TAMs towards an M2-like phenotype, promoting their involvement in immune escape and angiogenesis of tumor cells [45–48]. Therefore, M1 macrophages have the ability to inhibit the progression of tumors, while M2 macrophages are positively correlated with tumor advancement [49, 50]. Moreover, Kuo et al. have identified that the activation of oxidative-related pathways often results in the suppression of the immune functionality within the tumor microenvironment. This activation further induces the generation of reactive oxygen species (ROS), subsequently promoting the polarization of macrophages from the M1 to M2 phenotype. This, in turn, facilitates tumor growth and progression, exerting an adverse impact on patient prognosis [51, 52]. The activation of ROS is often accompanied by increased angiogenesis and the suppression of immune microenvironment functions. This further enhances the production of macrophage-derived interferon (IFN) and interleukin-6 (IL-6), thereby inhibiting immune responses within the tumor microenvironment [53, 54]. The oxidative-related pathways exhibit significant scores in both EPCs and the C0 subpopulation. Additionally, there is a notable negative correlation between the oxidative-related pathway scores and the GSEA immune pathway scores in C0. Consequently, we hypothesize that oxidative-related pathways play a crucial role in tumor progression, possibly establishing close associations with drug resistance and immune suppression Mouton’s research has similarly established the dependence of M2 macrophages on the tricarboxylic acid cycle and oxidative phosphorylation [55]. Consequently, we hypothesize that by modulating oxidative phosphorylation, not only can ovarian cancer cell resistance be improved, but the immune suppression within the tumor microenvironment can also be alleviated.

Ultimately, we have identified six prognostically relevant genes, with RPL23 exhibiting the highest coefficient value. RPL23, a ribosomal protein responsible for translating mRNA into proteins, has been implicated in cancer progression across various cancer types, including lung cancer, colorectal cancer, and hepatocellular carcinoma [56–58]. It has also been associated with increased drug resistance, promoting multidrug resistance in gastric cancer cells by inhibiting drug-induced apoptosis [59, 60]. We postulate that RPL23 plays a positive role in ovarian cancer progression, potentially linked to immune function and oxidative phosphorylation. However, further experimental validation is warranted. Kang and colleagues have also identified RPL23’s role in promoting ovarian cancer cell progression, suggesting that its high expression may facilitate ovarian cancer recurrence, aligning with our research findings [61].

Notably, the absence of integrated omics data, including metabolomics, ATAC-seq, or proteomics, represents a noteworthy deficiency in the current analysis. This limitation underscores the need for future research endeavors that encompass a more comprehensive multi-omics approach to enhance the depth and validity of our findings.

## CONCLUSIONS

In this study, we conducted a systematic exploration of single-cell data from ovarian cancer tissues, providing insights into the resistance mechanisms within EPCs and their association with the Oxidative Phosphorylation pathway. We further performed temporal trajectory analysis of various EPCs subtypes, highlighting the pivotal role of the central C0 subtype in immune cell functionality and correlation. Additionally, we constructed a prognostic model relevant to ovarian cancer, emphasizing the potential significance of the high-risk gene RPL23 and the Oxidative Phosphorylation pathway as crucial targets for ovarian cancer treatment and drug resistance. In conclusion, our discoveries unveil novel mechanisms related to drug resistance in ovarian cancer, paving the way for subsequent research. They also suggest another potential direction for targeting in ovarian cancer treatment and contribute to the construction of a new and effective prognostic model.

## Supplementary Material

Supplementary Figure 1
