# Supplementary material for: Investigating the mechanisms of drug resistance and prognosis in ovarian cancer using single-cell RNA sequencing and bulk RNA sequencing
Source: Aging (Albany NY). 2024 Mar 8;16(5):4736–58. doi: 10.18632/aging.205628 (PMC10968697; doi:10.18632/aging.205628)
Supplement: Supplementary Figure 1 [file aging-16-205628-s001.pdf]

SUPPLEMENTARY FIGURE

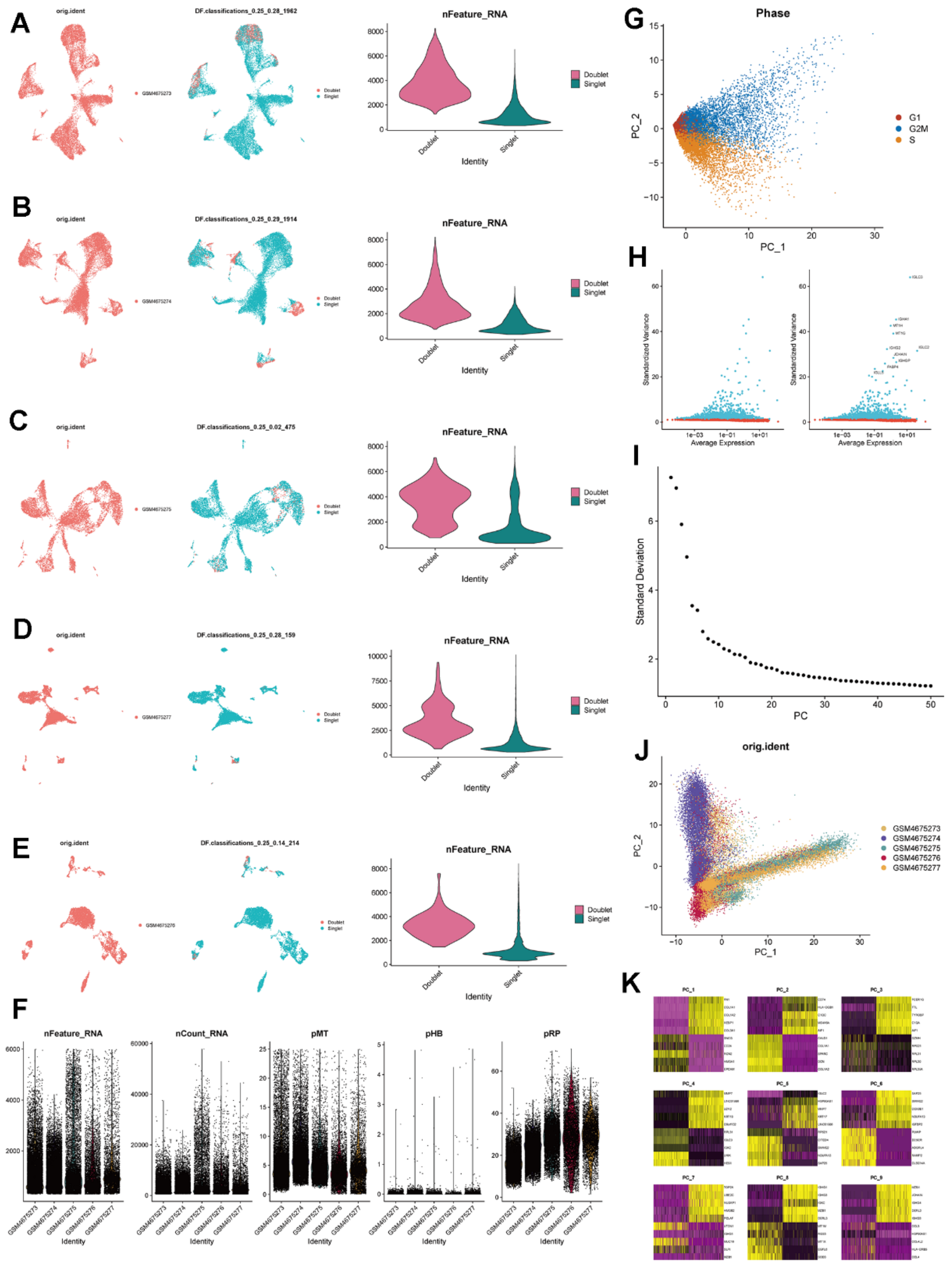

Supplementary Figure 1. Data control and standard procedures. (**A**–**E**) The dimplot and violin plots illustrate the distribution of double cells in each single-cell sample. (**F**) Violin plots of the filtered data. (**G**) Cell cycle distribution. (**H**) Distribution of highly variable genes. (**I**) Differences in each dimension. (**J**) Sample distribution in the first two principal components (PC1 and PC2). (**K**) Highly variable genes in each principal component (first 9 PCs).
